# Creating a community-based teaching clinic to support undergraduate and postgraduate medical education

**Published:** 2018-03-27

**Authors:** Elizabeth Wooster, Douglas Wooster, Axelle Pellerin, Rishie Seth, Jerry Maniate

**Affiliations:** 1 St. Joseph’s Health Centre (Toronto), Ontario, Canada; 2 OISE/University of Toronto, Ontario, Canada; 3 Wilson Centre, University of Toronto, Ontario, Canada; 4 Faculty of Medicine, University of Toronto, Ontario, Canada; 5 Argyle Tact Public Affairs; 6 The Ottawa Hospital, Ontario, Canada; 7 Faculty of Medicine, University of Ottawa, Ontario, Canada

## Introduction

While there has been a concurrent shift of clinical and training sites from traditional academic health sciences centres (AHSCs) to community settings, these training opportunities have not been well developed, nor grounded in appropriate educational theory, nor integrated into larger curricular objectives therefore leading to recent calls for change.^1-4^ At St. Joseph’s Health Centre (SJHC) there has been a long-standing history of medical education within the community hospital setting. In 2012 the establishment of the Department of Medical Education, Research and Scholarship (DMERS) meant a greater focus and additional resources could be directed towards creating supportive clinical learning environment for all learners. The underlying philosophy of this clinical environment has been the belief that the more experiences trainees were exposed to the more confidence they would develop.^5^

With the establishment of DMERS, an environmental scan of existing educational activities at SJHC (including undergraduate, postgraduate and continuing medical education) was conducted and opportunities for growth were identified. This led to the establishment of the Medical Education Teaching Clinic (METC) to address opportunities for optimization of education in the ambulatory care or outpatient setting underpinned by sound curricular development principles and connected to educational theories and practices.^6,7^ The METC was designed as a novel educational and research space focused in a community-based ambulatory care setting. The aim was to translate the experience of colleagues at a traditional AHSC setting to fit into the constraints and realities of a community-based hospital setting.^6,8^

## Rationale

Through a review of learner educational needs, local patient clinical needs and resource utilization and in collaboration with hospital clinical and support area leaders, clinic proposals were assessed. The development of the METC was intended to both provide trainees with unique high quality ambulatory experience in an authentic community-based hospital clinic setting and to address clinical needs within the hospital. One such clinic was the Vascular Teaching Clinic. The vast majority of admitted patients with vascular complications that do not require surgical intervention are managed by Internal Medicine specialists, and similarly, the vast majority of community-based non-admitted care provided to these patients is provided by Family Physicians. Interestingly, both groups of clinicians do not have formal Vascular Surgery exposure in their residency-training programs. Thus, this clinic exposure provides them with the opportunity to gain valuable knowledge and skills in the management of these complex patients under the guidance of Vascular Surgeons from an AHSC in Toronto.

## METC design, infrastructure, and resources

The METC was established in 2016 in order to create Engineered Educational Experiences (E^3^) - those training situations that are deliberately and purposefully created to maximize the trainees’ exposure to specific clinical situations and, ideally, result in an increase in trainee outcomes in high quality and high value educational experiences. Key features of E^3^ include: dedicated space for teaching and clinical activities, increased time scheduled for clinical activities, designated support staff, focused learning resources, low ratio of medical trainees and faculty, screening of referrals to diversify or consolidate clinic educational experiences, opportunities for hands-on learning and feedback, and improved setting for patient and family education.^6^

Practically, the METC provides four modern, furnished large clinic rooms with dedicated computer access that can easily accommodate the patient, family members, as well as staff physician/faculty and trainees. It was located in a vacated surgical unit with minimal renovation costs to make the space functional. Additionally, the METC provides debriefing and teaching spaces to support other educational activities including simulation and small-group teaching.

Dedicated staffing through DMERS was important to support all clinical and academic activities in the METC to ensure alignment with the vision and intention of the space. Clinical activities are additionally supervised by a Medical Director who liaises with clinicians to ensure high quality patient care is maintained in alignment with the hospital.

## Impact

Since its establishment, the METC has provided unique educational opportunities to over 75 medical students, physician assistant (PA) students, and residents from Family Medicine, Internal Medicine, and Surgery. Most importantly we have seen a positive impact of these opportunities on the education of our trainees while meeting the clinical needs of our patients closer to their home. In addition to the teaching clinics, the METC has been utilized to host regular educational sessions, such as trainee and staff orientation and educational research projects in simulation.

The early review of the METC noted positive feedback from medical trainees, who reported that these clinics addressed gaps in their current education by providing them with a chance to make connections between related areas of content, to think about patients more holistically and about the patient’s entire illness journey. Trainees also noted that there was substantial time for high quality teaching, observation, and feedback of clinical interactions by preceptors. Additionally, patients overwhelmingly noted that the medical team took time to explain their diagnoses and management in a way they could understand in this setting. In 2017, additional teaching clinics were established within the METC utilizing the same educational principles and logistics processes.

## Next steps

Prior to expanding the METC further, a structured evaluation project will be implemented to explore the experience of trainees, faculty as well as patients within the METC, and that of referring clinicians. We will also be exploring the utility of evaluating support staff experience within this space. The goal will be to rollout this evaluation project to include all ambulatory care settings within SJHC beyond the METC as a means of optimizing those clinical learning spaces.

## Summary

The ambulatory setting of SJHC, based on its community-focus, size, patient volumes and complexity, and interest in experiential learning provided an opportunity for innovations in medical education. We believe that the METC, underpinned by modest infrastructure investment and educational principles and theory, complements those opportunities experienced in traditional ambulatory care settings in AHSCs and is transferable to other settings. The METC is a locally developed example of an educational organizational structure that supports experiential learning and increases the opportunity for teaching and feedback for medical trainees.
